# Development and validation of a nomogram for predicting necrotizing pneumonia in children with refractory *Mycoplasma pneumoniae* pneumonia

**DOI:** 10.1186/s13052-025-02006-7

**Published:** 2025-05-21

**Authors:** Xiaoying Li, Lihua Zhao, Xiaojian Cui, Yongsheng Xu, Tongqiang Zhang, Wei Guo, Jing Ning

**Affiliations:** 1 Department of Pulmonology, Tianjin Key Laboratory of Birth Defects for Prevention and Treatment, Tianjin Children’s Hospital (Children’s Hospital of Tianjin University, Tianjin Pediatric Research Institute, Tianjin, 300134 China; 2 Department of Clinical Lab, Tianjin Key Laboratory of Birth Defects for Prevention and Treatment, Tianjin Children’s Hospital (Children’s Hospital of Tianjin University, Tianjin Pediatric Research Institute, Tianjin, 300134 China

**Keywords:** *Mycoplasma pneumoniae* pneumonia, Pulmonary necrosis, Necrotizing pneumonia, Nomogram, Prediction model

## Abstract

**Background:**

The early prediction of pulmonary necrosis in children with severe pneumonia improves patient prognosis and prevents complications. The aim of this study was to establish a linear model for predicting necrotizing pneumonia (NP) caused by *Mycoplasma pneumoniae* (MP) infection and to investigate the risk factors for lung necrosis in children with refractory *Mycoplasma pneumoniae* pneumonia (RMPP).

**Methods:**

A total of 536 children with RMPP were enrolled, including 95 with NP and 441 with nonnecrotizing pneumonia (NNP). A prediction model was built on 375 cases and validated on 161 cases, which were divided by random sampling in R software. Multivariate logistic regression was performed to determine optimal predictors and to establish a nomogram for predicting NP. The performance of the nomogram was evaluated by the area under the characteristic curve (AUC), calibration ability and decision curve analysis (DCA).

**Results:**

There were 315 (84.0%) NNP patients and 60 (16.0%) NP patients in the training group (*n* = 375) and 126 (78.3%) NNP patients and 35 NP patients (21.7%) in the validation group (*n* = 161). Multivariate logistic regression analysis identified 4 independent predictors that were used to construct a nomogram for predicting NP in children with RMPP, namely, fever duration (AOR = 1.475; 95% CI 1.296–1.678; *P* < 0.001), WBC count (AOR = 1.149; 95% CI 1.073–1.231; *P* < 0.001), IL-6 concentration (AOR = 1.007; 95% CI 1.002–1.013; *P* = 0.007) and D-dimer concentration (AOR = 1.361; 95% CI 1.121–1.652; *P =* 0.002). The area under the curve (AUC) of the nomogram was 0.899 (95% CI, 0.850–0.947) in the training set and 0.920 (95% CI, 0.874–0.966) in the validation set, indicating a good fit. The calibration plot and Hosmer‒Lemeshow test indicated that the predicted probability had good consistency with the actual probability in the training (*P* = 0.439) and validation (*P* = 0.526) groups. The DCA curve demonstrated a significantly better net fit in the model.

**Conclusions:**

We developed and validated a nomogram model for predicting RMPP-associated NP in its early clinical stages based on fever duration, WBC count, IL-6 and D-dimer concentration. This four-risk factor model may assist physicians in predicting NP induced by RMPP.

**Supplementary Information:**

The online version contains supplementary material available at 10.1186/s13052-025-02006-7.

## Introduction

*Mycoplasma pneumoniae* (MP) is one of the most important pathogens that causes community-acquired pneumonia in school-age children and adolescents [1, 2]. *Mycoplasma pneumoniae* pneumonia (MPP) was previously considered a self-limiting disease with a good prognosis [3]. However, some patients develop refractory *Mycoplasma pneumoniae* pneumonia (RMPP) even after seven or more days of macrolide treatment [4]. Recently, an increasing number of children with RMPP have been reported. Having RMPP may result in significantly decreased quality of life for the child and impose economic burdens on both families and society. The complications of patients with RMPP are relatively serious and may include necrotizing pneumonia (NP), persistent atelectasis, obliterated bronchitis, and bronchiectasis [5, 6].

NP is essentially a pathological diagnosis characterized by structural destruction, liquefaction, and necrosis of the lung tissue, indicating inflammatory infiltration. Patients may experience intrapulmonary vascular embolism with multiple cavities involving lung segments or lobes. The imaging features of the NP include at least one thin-walled cavity in the infected area with no edge enhancement on contrast-enhanced chest computed tomography (CT) [7]. NP is common in children with *Staphylococcus aureus* pneumonia but rare in those with MPP [8]. NP may suddenly manifest as liquid pneumothorax and dyspnea, which can be life-threatening. Therefore, clinicians must monitor patients with RMPP closely to prevent or lessen the severity of NP.

To the best of our knowledge, no previous studies have developed a nomogram to indicate potential risk factors for predicting NP in patients with RMPP. An effective nomogram may aid in early diagnosis and improve the clinical treatment of NP.

Therefore, in this study, we retrospectively analyzed the clinical characteristics, laboratory results, and imaging features of children with RMPP and NP. Subsequently, a model was developed to predict the risk of NP in children with RMPP. The discrimination, calibration, and clinical effectiveness of the prediction model were verified and evaluated.

## Methods

### Participants/aim/design/setting

Patients between the ages of 1 and 16 years who were diagnosed with RMPP at Tianjin Children’s Hospital (Tianjin, China) between January 2018 and December 2019 were enrolled. A total of 536 children with RMPP were enrolled, including 95 with NP and 441 with nonnecrotizing pneumonia (NNP). The 536 patients’ data were divided into a training group and a validation group by random sampling in R software. The clinical, imaging, and laboratory data of the included patients were retrospectively obtained from hospital medical records.

The diagnosis of MPP is based on the following: symptoms and signs indicative of pneumonia, including fever, expectoration, or wheezing and abnormal lung auscultation; chest imaging showing inflammatory infiltration; and an MP-immunoglobulin M (IgM) titer ≥ 1:160 or four-fold rising titer in acute and convalescent serum specimens or positive results for MP-RNA in throat swabs and bronchoalveolar lavage fluid [9].

The diagnostic criteria for RMPP are persistent fever and/or further imaging deterioration after seven days of early macrolide treatment [4].

The inclusion/diagnostic criteria for NP included normal previous lung imaging, a definitive diagnosis of MPP, and early lung CT suggesting solid lung changes or combined pleural effusion. With disease progression, the criteria may include liquefied necrosis of the lung tissue in the original solid lung area; multiple small, thin-walled cavities filled with gas or fluid, some of which may fuse to form larger cavities; and contrast-enhanced chest CT showing no marginal enhancement [7, 10].

The exclusion criteria for this study were underlying diseases, such as congenital heart and lung diseases; immunodeficiency diseases; a history of foreign body aspiration diagnosed using fiberoptic bronchoscopy or chest CT; Patients followed for motor disabilities such as cerebral palsy; coinfection with other pathogens or infection at other sites; and missing retrospective data from medical records.

This study was approved by the Ethics Committee of Tianjin Children’s Hospital. Verbal consent was obtained from all participants/families, and written informed consent was obtained before enrollment.

### Data collection

The clinical data of patients in the acute infection stage, including sex, age, clinical symptoms and signs, fever duration, extrapulmonary and pulmonary complications, and treatment, were retrospectively collected. All patients were examined using lung CT to determine the presence of focal or segmental lesions, pleural effusion, and the area of the lung lobe affected by lesions. In addition, patients underwent diagnostic testing for complete blood count, blood biochemistry, inflammatory indicators, MP-RNA in sputum/bronchoalveolar lavage fluid, and other pathogenic microorganisms. Data from CT imaging and other aforementioned test results were collected and recorded. Furthermore, fever duration during hospitalization, the dose and duration of glucocorticoid treatment, and the presence or absence of cavities and necrosis on chest imaging after treatment were recorded.

### Statistical methods

#### Variable selection and model construction

The 536 patients’ data were divided into a training group (70% of the data, 375 patients) and a validation group (30% of the data, 161 patients) by random sampling in R software with the “sample()” function.

The data of all 375 patients in the training group were analyzed for variable selection and risk prediction. Multivariate logistic regression analysis was used to construct a nomogram model to predict the occurrence of NP. The selected independent predictors (*P* < 0.05) were evaluated by multivariate logistic regression, applied to establish the risk prediction model and presented with a nomogram. The values of the selected predictors on the corresponding axis were marked from the nomogram. A vertical line is drawn from the value to the top lines, and the corresponding points are obtained. The sum of these points was located on the “Total Points” axis; subsequently, a line was drawn downward to project on the bottom scales, which determined the possibility of NP. Thereafter, the prediction model was validated in the validation cohort.

#### Validation of the nomogram

The performance of the prediction model was evaluated in terms of discrimination ability, calibration ability and clinical value. The discrimination ability was evaluated through the area under the receiver operating characteristic curve (AUC), and a calibration plot accompanied by the Hosmer‒Lemeshow test was generated to assess the calibration ability. The model was validated using the bootstrap method with 1000 resamples to quantify any overfitting. Decision curve analysis (DCA) was applied to evaluate the clinical utility of the nomogram based on its net benefits at different threshold probabilities.

### Statistical analysis

Continuous variables are expressed as the means ± standard deviations (SDs) or medians (interquartile ranges) and were assessed by independent group t tests or Mann‒Whitney U tests according to the type of data distribution. Categorical variables are expressed as percentages (%) and were assessed by chi-squared tests. Statistical analysis was carried out using SPSS 26.0, and R software (version 4.0.5, http://www.r-project.org) was used to construct all the graphics based on the R packages “foreign”, “rms”, “ggplot2”, “pROC” and “glmnet”. Variables significant (*p* < 0.05) at univariate analysis were included in the multivariable model. *P* < 0.05 was considered to indicate statistical significance.

## Results

### Patient characteristics

A total of 536 children with RMMP met the inclusion criteria, and 95 patients were diagnosed with NP. The data were randomly divided into a training group (*n* = 375) and a validation group (*n* = 161). There were 60 (16%) and 35 (21.7%) patients with NP in the training and validation groups, respectively. The baseline clinical data and proportion of NP patients were similar between the training and validation groups (Table 1).

**Table 1:** The variables of the training and validation cohorts

	Train group(*n* = 375)	Test group(*n* = 161)	*P*
Age (years)	6.3 ± 2.7	6.5 ± 3.1	0.513
Male, n (%)	187 (49.9%)	81 (50.3%)	0.925
NP	60 (16%)	35 (21.7%)	0.111
fever (n, %)	373 (99.5%)	160 (99.4%)	0.901
length of fever (day)	9.9 ± 3.7	9.8 ± 3.9	0.849
Total length of hospital stay (day)	8.7 ± 3.8	9.3 ± 5.0	0.127
Hyoxemia (n, %)	23 (6.1%)	10 (6.2%)	0.973
Plastic bronchitis (%)	53(14.1%)	26(16.1%)	0.546
• Macrolide should be applied within 5 days (n, %)	246 (65.6%)	113 (70.2%)	0.301
Hormone (n, %)	243 (64.8%)	106 (65.8%)	0.817
Gamma globulin (n, %)	23 (6.1%)	12 (7.5%)	0.571
WBC (×10^9^/L)	9.3 ± 5.0	9.1 ± 4.8	0.696
NE (%)	65.7 ± 14.3	66.0 ± 12.5	0.825
PLT (×10^9^/L)	314.7 ± 121.9	311.3 ± 109.4	0.762
CRP (mg/L)	29.9 (14.6–53.2)	26.3 (12.0-45.8)	0.248
PCT (ng/ml)	0.13 (0.07–0.23)	0.13 (0.08–0.29)	0.612
IL-6 (pg/ml)	24.2 (14.5–48.9)	25.1 (14.9–58.1)	0.539
AST (U/L)	29.0 (23.0–39.0)	32.0 (25.0–42.0)	0.061
ALT (U/L)	14.0 (11–21)	14.0 (11–24)	0.453
CK (U/L)	88 (59–139)	91 (58–188)	0.248
CKMB (U/L)	4 (3–4)	4 (3–5)	0.921
LDH (U/L)	387 (309–425)	408 (322–527)	0.674
FER	145.9 (104-252.1)	149.5 (99.3-264.2)	0.878
APTT (s)	30 (26.9–34.2)	29.8 (26.2–34.0)	0.348
FG (g/l)	4.1 (3.6–4.5)	4.1 (3.5–4.6)	0.610
D-Dimer (mg/L)	0.2 (0.1–0.6)	0.2 (0.1–0.7)	0.992
IgE (IU/L)	92.1 (41.3-215.4)	109.1(37.9-276.8)	0.551
Multiple lung lobes were involved (n, %)	252 (67.2%)	107 (66.5%)	0.867
Pulmonary atelectasis (n, %)	102 (27.2%)	44 (27.3%)	0.975
Pleural thickening (n, %)	245 (65.3%)	105 (65.2%)	0.979
Pleural effusion (n, %)	105 (28.0%)	47 (29.2%)	0.779

### Predictors of NP

In the training group, children with NP were younger (5.6 ± 3.2 vs. 6.5 ± 2.6, *P* = 0.032); had a shorter fever (14.2 ± 5.4 vs. 9.1 ± 2.6, *P* < 0.001); had a shorter length of stay (14.7 ± 4.3 vs. 7.6 ± 2.4, *P* < 0.001); had a greater incidence of multiple lobe involvement (83.3% vs. 64.1%, *P* = 0.004), pleural thickening (80.0% vs. 62.5%, *P* = 0.009), pleural effusion (51.7% vs. 23.5%, *P* < 0.001) and IVIG treatment (18.3% vs. 3.8%, *P* < 0.001); had a greater median WBC (13.7 vs. 8.4 × 10^9^/L, *P* < 0.001), N%(neutrophils percentage) (73.3% vs. 64.3%, *P* < 0.001), PLT (364.9 vs. 305.1 × 10^9^/L, *P* = 0.005), CRP (60.0 vs. 24.3 mg/L, *P* < 0.001), and PCT (0.18 vs. 0.12 ng/ml, *P* = 0.002). There were no significant differences in AST, ALT, CKMB, IgE, sex ratio, incidence of fever, hypoxemia, application of macrolides within 5 days, pulmonary atelectasis or steroid treatment (Table 2).

**Table 2:** The clinical, laboratory and radiological features of RMPP patients in the training group

	Train (*n* = 375)	NNP (*n* = 315)	NP (*n* = 60)	*P*
Age (years)	6.3 ± 2.7	6.5 ± 2.6	5.6 ± 3.2	0.032
length of fever (day)	9.9 ± 3.7	9.1 ± 2.6	14.2 ± 5.4	< 0.001
Total length of hospital stay (day)	8.7 ± 3.8	7.6 ± 2.4	14.7 ± 4.3	< 0.001
Male, n (%)	187 (49.9%)	153 (48.6%)	34 (56.7%)	0.325
fever (n, %)	373 (99.5%)	313 (99.4%)	60 (100%)	0.536
Hyoxemia (n, %)	23 (6.1%)	19 (6%)	4 (6.7%)	0.851
Plastic bronchitis (%)	53 (14.1%)	46 (14.6%)	7 (11.7%)	0.550
Macrolide should be applied within 5 days (n, %)	246 (65.6%)	208 (66%)	38 (63.3%)	0.687
Multiple lung lobes were involved (n, %)	252 (67.2%)	202 (64.1%)	50 (83.3%)	0.004
pulmonary atelectasis (n, %)	102 (27.2%)	83 (26.3%)	19 (31.7%)	0.396
pleural thickening (n, %)	245 (65.3%)	107 (62.5%)	48 (80.0%)	0.009
pleural effusion (n, %)	105 (28.0%)	74 (23.5%)	31 (51.7%)	< 0.001
Hormone (n, %)	243 (64.8%)	202 (64.1%)	41 (68.3%)	0.532
Gamma globulin (n, %)	23 (6.1%)	12 (3.8%)	11 (18.3%)	< 0.001
Time to necrosis (n, %)			15 (10–25)	
WBC (×10^9^/L)	9.3 ± 5.0	8.4 ± 4.1	13.7 ± 6.7	< 0.001
NE (%)	65.7 ± 14.3	64.3 ± 14.1	73.3 ± 12.9	< 0.001
PLT (×10^9^/L)	314.7 ± 121.9	305.1 ± 113.1	364.9 ± 151.7	0.005
CRP (mg/L)	29.9 (14.6–53.2)	24.3 (11.9–42.0)	60 (51.5–76.2)	< 0.001
PCT (ng/ml)	0.13 (0.07–0.23)	0.12 (0.07–0.23)	0.18 (0.10–0.76)	0.002
IL-6 (pg/ml)	24.2 (14.5–48.9)	22.7 (14.0-41.3)	59.6 (22.3-160.4)	< 0.001
AST (U/L)	29.0 (23.0–39.0)	29.0 (23.0–38.0)	30.5 (23.0–48.0)	0.306
ALT (U/L)	14.0 (11–21)	14.0 (11–20)	14.0 (11.3–24.5)	0.180
CK (U/L)	88 (59–139)	93 (62.0-143.0)	64.5 (44.5-115.8)	0.001
CKMB (U/L)	4 (3–4)	4 (3–4)	4 (4–6)	0.200
LDH (U/L)	387 (309–425)	378 (305–494)	460 (326-639.8)	0.001
FER	145.9 (104-252.1)	135.7 (97.4–214.0)	245.4 (140.6–429)	< 0.001
APTT (s)	30 (26.9–34.2)	29.7 (26.7–34.0)	32.5 (29.0-36.1)	0.025
FG (g/l)	4.1 (3.6–4.5)	4.0 (3.5–4.4)	4.5 (3.7–4.8)	0.001
D-Dimer (mg/L)	0.2 (0.1–0.6)	0.2 (0.1–0.4)	1.3 (0.4–5.3)	< 0.001
IgE (IU/L)	92.1 (41.3-215.4)	88.2 (40.3-222.8)	137.9 (44.9-212.8)	0.351

Multivariate logistic regression analysis revealed that fever duration ( adjusted OR, 1.475; 95% CI 1.296–1.678; *P* < 0.001), the median WBC count (AOR, 1.149; 95% CI 1.073–1.231; *P* < 0.001), the IL-6 concentration (AOR, 1.007; 95% CI 1.002–1.013; *P* < 0.05), and the median WBC count (AOR, 1.361; 95% CI 1.121–1.652; *P* < 0.05) were independent predictors of NP (Table 3).

**Table 3:** Univariate and multivariate logistic regression for predicting RMPP-associated NP in the training group

	Exp (B)	95% CI	*P*	Exp (B)	95% CI	*P*
Age	0.892	0.804–0.991	0.033			
length of fever	1.526	1.357–1.715	< 0.001	1.475	1.296–1.678	< 0.001
Multiple lung lobes were involved	2.797	1.366–5.729	0.002	2.512	0.888–7.111	0.083
pleural thickening	2.396	1.223–4.694	0.011	2.343	0.890–6.169	0.085
pleural effusion	3.481	1.970–6.152	< 0.001			
WBC (×10^9^/L)	1.199	1.133–1.269	< 0.001	1.149	1.073–1.231	< 0.001
NE (%)	1.061	1.034–1.089	< 0.001			
PLT (×10^9^/L)	1.004	1.001–1.006	0.001			
CRP (mg/L)	1.019	1.012–1.027	< 0.001			
PCT (ng/ml)	1.570	1.146–2.149	0.005			
IL-6 (pg/ml)	1.012	1.007–1.017	< 0.001	1.007	1.002–1.013	0.007
CK (U/L)	0.998	0.995–1.001	0.121			
LDH (U/L)	1.002	1.001–1.003	0.005			
FER	1.002	1.001–1.003	< 0.001			
APTT (s)	1.048	1.003–1.094	0.035			
FG	1.592	1.190–2.130	0.002			
D-Dimer (mg/L)	1.549	1.321–1.817	< 0.001	1.361	1.121–1.652	0.002

### Development and validation of the NP-predicted nomogram

**Fig. 1:**
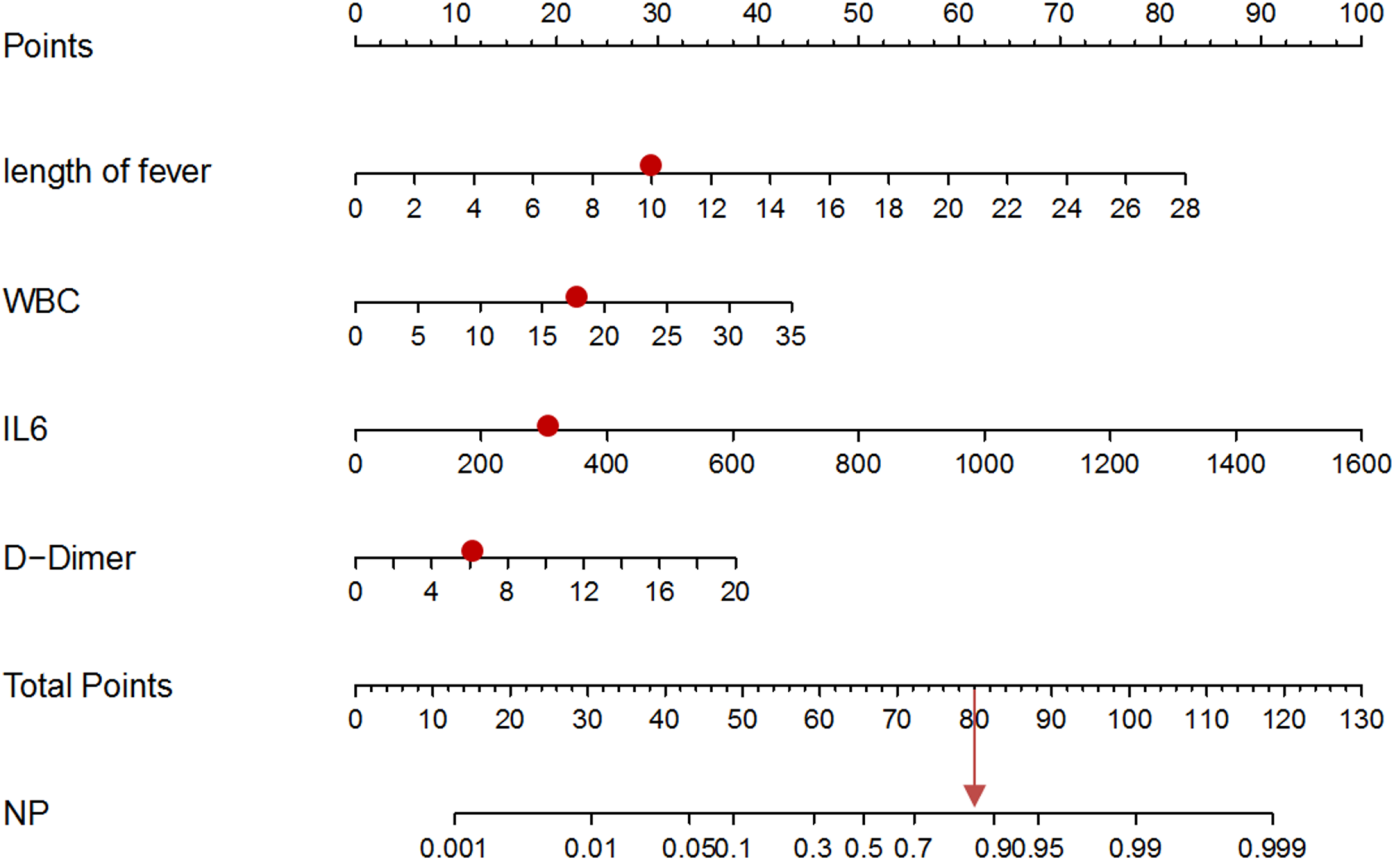
A nomogram to predict NP among children with RMPP was constructed based on 4 independent predictors. The values of these included factors are marked on the corresponding axes. A vertical line is drawn from the value to the top lines, and the corresponding points are obtained. Then, the points from each variable value are summed. Locate the sum on the total points scale and project it vertically on the bottom axis to obtain the NP risk

The 4 variables identified by multivariate regression analysis were applied to establish the risk model and are presented with a nomogram (Fig. 1): duration of fever, WBC, IL-6 and D-dimer levels. The total points based on the sum of the points for each predictor in this nomogram were associated with the risk of NP. As an example, to better explain the nomogram model, if the subject with RMPP has a fever for 10 days (approximately 28 points), a WBC of 17.5 × 10^9^/L (22 points), an IL-6 level of 300 pg/ml (18 points) and a D-dimer level of 6 mg/L (12 points), the probability of NP is estimated to be approximately 82%.

**Fig. 2:**
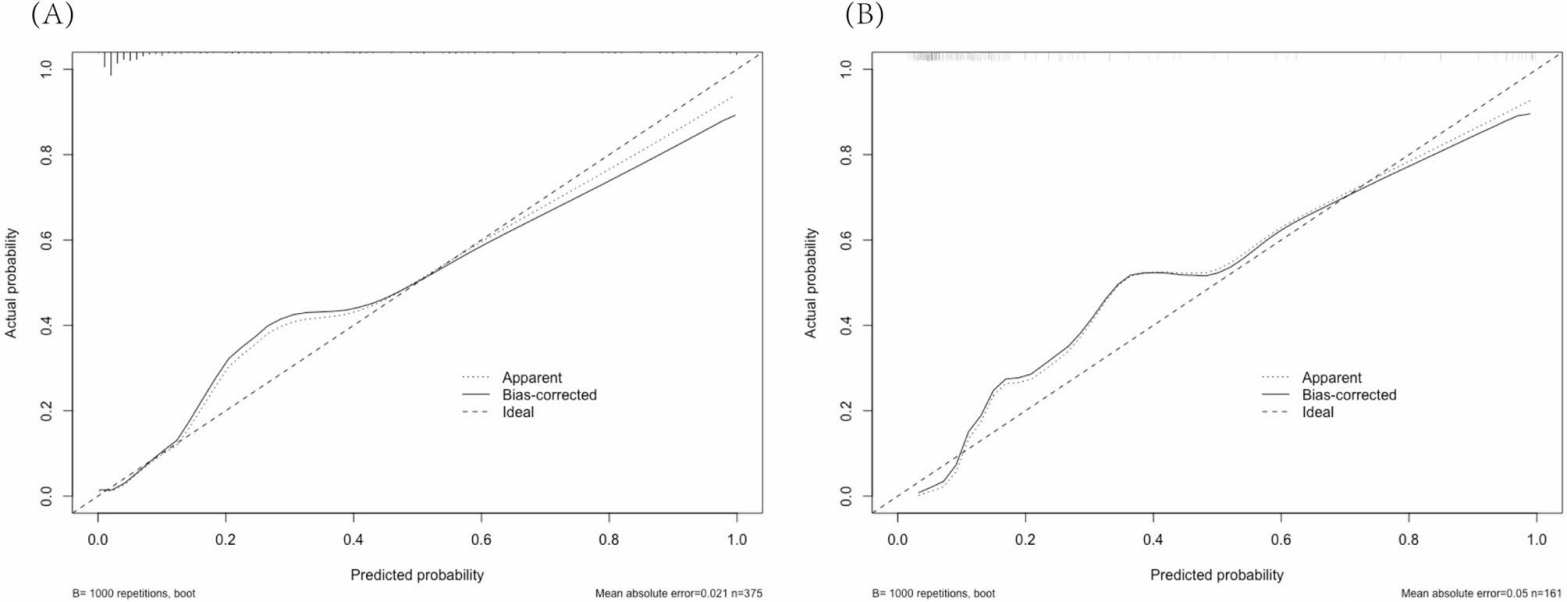
Calibration plot of the NP risk nomogram in the development cohort (**A**) and validation cohort (**B**). The ideal outcome (dashed line), the observed outcome (fine dashed line), and the bias-corrected outcome (solid line) are depicted

**Fig. 3:**
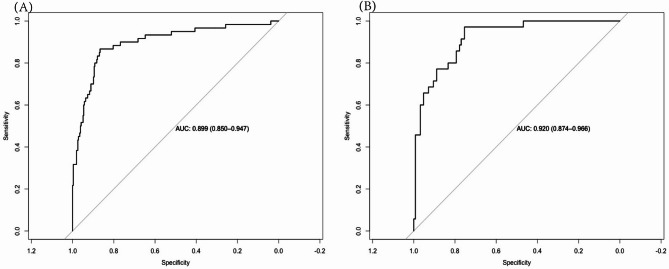
The ROC curves of the nomogran from the training dataset (**A**) and the validation dataset (**B**). ROC: receiver operating characteristic

By internal bootstrap validation with 1000 resamples, the mean area under the curve (AUC) of the nomogram based on the training group was 0.899 (95% CI, 0.850–0.947) (Fig. 2-A), with good discrimination ability for predicting NP cases in children with RMPP. Furthermore, the calibration plot (Fig. 3-A) and Hosmer‒Lemeshow test (*P* = 0.439) of the prediction model showed good consistency between the predicted probability and actual probability.

**Fig. 4:**
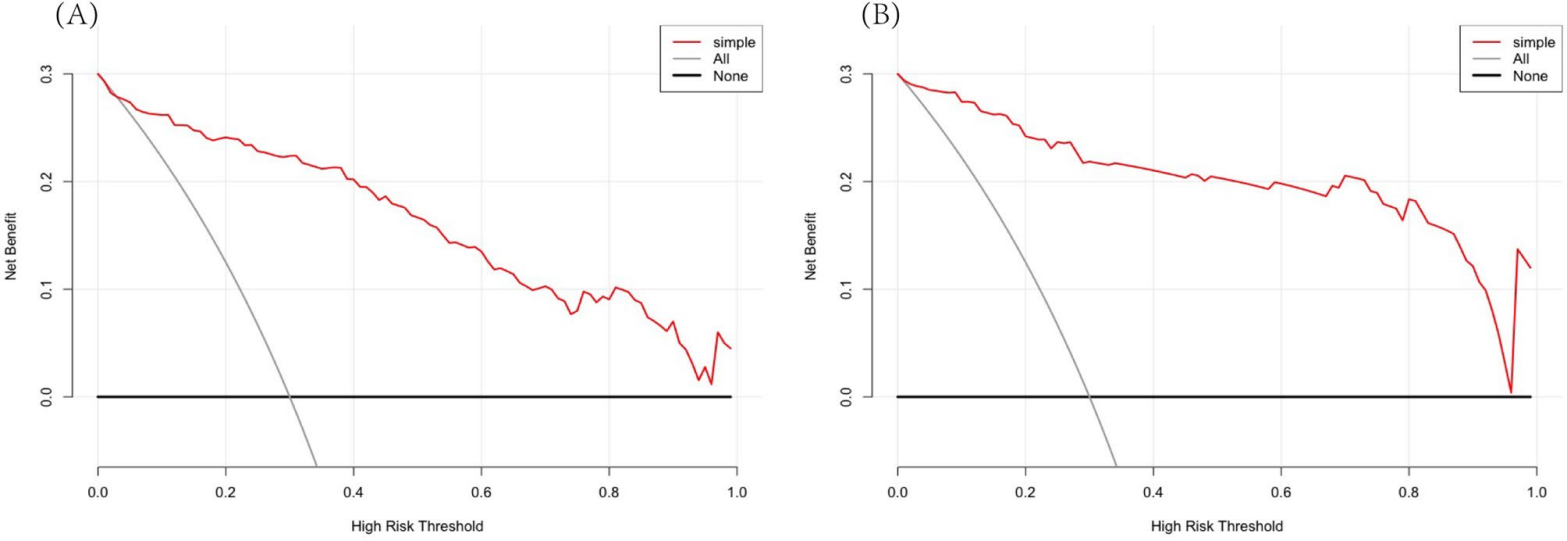
Decision curve analysis of the NP risk nomogram. The y-axis represents the net benefit. The black solid line represents the assumption that all patients had NNP. The gray solid line represents the assumption that all patients had NP. The red solid line represents the risk nomogram. (**A**) The training dataset and (**B**) the validation dataset

The accuracy of the nomogram in the validation cohort was similar to that in the training cohort, with an AUC of 0.920 (95% CI, 0.874–0.966) (Fig. 2-B). The calibration plot (Fig. 3-B) and Hosmer‒Lemeshow test (*P* = 0.526) showed that the prediction model fit well in the validation dataset. We applied a DCA curve to evaluate the clinical value of the prediction model. The DCA curve showed obvious net benefits of the predictive nomogram, which were significantly greater than those of the two extreme cases (Fig. 4).

## Discussion

*MP* is a common pathogen that causes community-acquired pneumonia in children. Recent studies have focused on the risk factors for pulmonary complications in patients with RMPP, such as BO (bronchiolitis obliterans), plastic bronchitis, bronchopleural fistula, persistent atelectasis, and NP. The diagnostic standard for NP is based on chest CT characteristics. During the course of NP, pulmonary consolidations initially manifest; approximately one week later, low-density lesions emerge within the consolidations. Subsequently, multiple cavities develop in the lung tissue. Patients with NP exhibit fluid pneumothorax and dyspnea, which are likely to occur following gentle or strenuous exercise, and NP can progress and become life threatening if not promptly diagnosed and treated. Therefore, we decided that developing a model to predict and diagnose NP early was of paramount importance. We believe that it may play a role in preventing the occurrence of serious complications. Using multivariate logistic regression analysis, we identified four factors associated with the risk of pulmonary necrosis caused by pediatric RMPP: fever duration, white blood cell (WBC) count, and elevated interleukin-6 (IL-6) and D-dimer levels. The nomogram model showed good discrimination, calibration, and clinical application.

Fever is closely associated with inflammatory responses in the body. When the body temperature increases, the basal metabolic rate increases, and a series of symptoms, such as increased heart rate, tachypnea, and dehydration, may occur. These responses may further alter immune defense mechanisms and lead to more serious complications. Currently, persistent high fever is generally considered to be related to the excessive inflammatory response caused by MP infection. The pathogenenis of *M. pneumonia* necrotizing pneumonia (MPNP) is not clearly clarified and are various, however the excessive inflammatory response contributes greatly to the progression of pulmonary necrosis [11]. The stronger the immune response, the more serious the organ damage. Regarding clinical characteristics, our study revealed that all children with NP had a long fever duration (i.e., more than 12 days). Similarly, a study by Wang et al. showed that all included patients with RMPP developed a long duration of fever before admission (mean duration of 13.2 ± 6.8 days) [12]. Another study reported an average fever duration of 21.0 ± 8.9 days among 25 children with *M. pneumonia* necrotizing pneumonia (MPNP) [13]. A receiver operating characteristic (ROC) curve showed that a critical value for diagnosing pulmonary necrosis is a fever duration of 11.5 days; this phenomenon can be used to predict MPNP [14]. Consistent with that finding, the present study showed that children in the NP group had a significantly longer fever duration than did those in the control/non-NP group (14.2 ± 5.4 vs. 9.1 ± 2.6, respectively). Upon further analyses, we included long fever duration as a high-risk factor in the nomogram. Therefore, fever duration can be used as an independent predictor of NP in children with RMPP. This has some value for the early prediction of MPNP because longer fever durations lead to greater damage to the lung parenchyma [15].

The specific pathogenesis of MPNP has not been determined; however, two explanations have been proposed: (1) direct damage from MP and its secreted toxins and (2) an excessive immune response, leading to secondary lung damage after MP infection. The combination of these two mechanisms results in local vasculitis or thrombosis, eventually leading to vascular occlusion. The present study showed that D-dimer levels could also be used as an independent risk factor for the development of RMPP in patients with NP. D-Dimer is a degradation molecule produced during fibrin hydrolysis. It can be used as a specific marker for the fibrinolytic system and is one of the most significant indicators for monitoring inflammation and severe infection [16]. Cerda-Mancillas et al. [17] reported a significant relationship between D-dimer levels and the severity of community-acquired pneumonia in children, which is consistent with other findings [18]. Another study revealed that D-dimer levels (> 1.3675 mg/L) were independent predictors of NP in children with MPP [19]. Our study also revealed that D-dimer levels in children with MPNP were significantly greater than those in the control group (0.8 vs. 0.2 mg/L, respectively). D-Dimer levels also showed good predictive performance in our nomogram model for the occurrence of NP in children. Based on the aforementioned findings, we believe that the D-dimer level is an effective predictor of NP development in children with RMPP. This suggests that, in clinical practice, children with RMPP should be treated with low-molecular-weight heparin anticoagulant therapy based on their D-dimer levels. However, due to the small sample size of our study, the predictive cutoff value has certain limitations, and more multicenter and large-sample clinical data studies are needed to validate these findings for clinical application.

MP can activate or induce mast cells and lymphocytes to produce cytokines such as interleukin, interferon, and tumor necrosis factor-α that produce strong inflammatory responses. In addition, MP forms circulating immune complexes with tissue antigens in the human body, leading to autoimmune reactions. Stronger immune and cytokine responses lead to more serious organ damage and disease [20]. Relevant studies have reported a correlation between interleukin levels and NP. Interleukins are key factors in the pathogenesis of NP. They form pores in the cell and mitochondrial membranes of neutrophils and macrophages, causing cell lysis and apoptosis, which in turn promotes the release of inflammatory mediators [10]. Zhou et al. [21] found that the IL-6 levels of children with MPP in the NP group were significantly greater than those in the non-NP group (47.9 vs. 29.2 pg/mL, respectively). The IL-6 levels (59.6 vs. 23.7 pg/mL) in the present study are similar to those reported in previous studies. However, our nomogram model confirmed that the level of IL-6, which is the cause of host immune disorders, is an appropriate independent risk factor for predicting the occurrence of NP in children. This suggests that when IL-6 levels are elevated, clinicians should suspect NP, and hormones and immunoglobulins should be administered promptly as appropriate.

Blood cell analysis is one of the most common and essential laboratory tests used in clinical practice. We found that the WBC counts were markedly increased in patients with RMPP and NP. A study conducted by Seo et al. involving 830 patients demonstrated that the WBC count can potentially increase to 14.97 × 10^9^/L in patients with NP, which is significantly greater than that reported in a non-NP group (10.13 × 10^9^/L) [22]. Another study on children with MPP showed that predicting NP using a WBC count of 15.1 × 10^9^/L had a sensitivity of 0.5 and a specificity of 0.929 [23]. This indicates that the WBC count can be used as an independent predictor of NP. Zheng et al.’s study showed that pediatricians should consider NP in patients with a WBC of 12.3 × 10^9^/L and a neutrophil ratio of 73.9% [19]. The present study also revealed a significantly greater WBC count in children with NP than in children without NP (13.2 vs. 7.4 × 10^9^/L, respectively). Our constructed nomogram model suggested that for children with a WBC count greater than 13.2 × 10^9^/L, physicians should consider using glucocorticoids to inhibit the inflammatory response for active anti-infective treatment to prevent or delay the process of pulmonary necrosis. In addition, When there is a significant increase in WBC count in children with RMPP, pulmonary imaging changes should be closely monitored for the early detection of pulmonary necrosis.

Furthermore, pleural thickening and the occurrence of pulmonary complications, such as pleural effusion, cannot currently be used as independent factors to predict NP. A previous study of 135 children with MPP enrolled over two years revealed no significant difference in pleural thickening or effusion between the NP and non-NP groups [10], which was consistent with our findings. Furthermore, consistent with findings reported in other study [19], the C-reactive protein (CRP) levels in children with NP was significantly higher than those in the control group (62.7 vs. 38.67 mg/L). However, the CRP differences between the NP and control groups (60.0 vs. 24.3 mg/L, respectively) indicated poor predictive performance of our model. Therefore, the CRP level was not included in the nomogram. However, further studies are required to verify the correlations between CRP levels and NP. In accordance with a prior study that employed multivariate logistic analysis to assess factors associated with NP, CRP and lactate dehydrogenase (LDH) levels differed significantly between patients with and without NP (74.0 vs. 38 mg/L, respectively, and 360 vs. 271 U/L, respectively). These findings underscore the importance of CRP and LDH levels in predicting the occurrence of NP [24]. Similarly, other studies concluded that the critical values of CRP and LDH for predicting NP in children are 125.1 mg/L and 353.5 U/L, respectively [25]. NP should be considered in children with large consolidations on lung imaging with concomitant persistent high fever; significantly increased levels of CRP, procalcitonin, and other inflammatory indicators; and pleural effusion. These laboratory and other indices cannot be ignored in predicting the occurrence of NP and should be combined with clinical and imaging manifestations and other laboratory results for comprehensive analysis. Validating these recommendations requires more reported cases and more dynamic evaluations of children in the future.

Children with NP have a long fever duration and significantly increased levels of inflammatory markers. Our nomogram confirmed that fever duration and WBC, IL-6, and D-dimer levels are risk factors for pneumonia complicated by NP. With a fever longer than 12 days, inflammatory markers continue to increase, and the patient reaches a hypercoagulable state. Chest CT should be performed promptly to determine the presence or extent of lung necrosis, and active anti-inflammatory treatment should be administered to decrease the occurrence of pulmonary necrosis and improve prognosis.

In patients with RMPP, persistent high fever, along with marked elevations in WBC counts, IL- 6 levels, and D - dimer values may be the indicator of NP and pulmonary imaging changes should be closely monitored for the early detection of pulmonary necrosis. The study constructed a simple and practical clinical predictive nomogram, which shows good clinical practicability both in the development and validation datasets. It may be a very suitable tool for the early identification of NP, thereby making contribution to timely intervention and appropriate treatment.

However, this study has several limitations that must be considered. First, some patients may have been coinfected with other pathogens that were undetectable using our methods. Secondly, we did not incorporate patients younger than 1 years old which may need further study. In addition, this was a single-center study, and all research data came from one medical center. This limits the generalizability of our results to other institutions and fields. Multicenter studies with large sample sizes need to be developed to validate our results for clinical applicability. Finally, our prediction model was based on a retrospective study, and individuals with incomplete data were excluded, which may have led to selection bias.

## Conclusions

We successfully established and validated a nomogram for predicting RMPP-associated NP in the early clinical stages based on 4 variables commonly measured on admission to the hospital, including fever duration, WBC count, IL-6 and D-dimer concentration. Our simple and convenient nomogram may assist physicians in identifying risk factors that could lead to NP in patients with RMPP. This may promote enhanced diagnostic strategies and prompt treatment to improve the outcomes of patients with RMPP.

## Electronic supplementary material

Below is the link to the electronic supplementary material.

Supplementary Material 1

## Data Availability

The datasets used and/or analyzed during the current study are available from the corresponding author upon reasonable request.

## Abbreviations

ALT: Alanine aminotransferase
AST: Aspartate aminotransferase
BO: Bronchiolitis obliterans, CK, creatine kinase
CKMB: Creatine kinase isomer-MB
CRP: C-reactive protein
CT: Computed tomography
DCA: Decision curve analysis
IL-6: Interleukin-6
LDH: Lactate dehydrogenase, Mycoplasma pneumoniae
MPNP: Mycoplasma pneumoniae necrotizing pneumonia
MPP: Mycoplasma pneumoniae pneumonia
N%: Neutrophils percentage
NP: Necrotizing pneumonia
RMPP: Refractory Mycoplasma pneumoniae pneumonia
ROC: Receiver operating characteristic, WBC, white blood cell
