# Supplementary material for: Development and validation of a nomogram for predicting necrotizing pneumonia in children with refractory Mycoplasma pneumoniae pneumonia
Source: Ital J Pediatr. 2025 May 21;51:150. doi: 10.1186/s13052-025-02006-7 (PMC12096616; doi:10.1186/s13052-025-02006-7)
Supplement: Supplementary file 1 — Supplementary Material 1 [file 13052_2025_2006_MOESM1_ESM.docx]

Response to reviewers

Dear Prof. Elena Bozzola：

We sincerely thank all of you for the constructive comments and we totally agree with your suggestions which might be of great help to improve our manuscript. We have revised it accordingly and would like to resubmit our revised manuscript for your consideration.

The revised parts are highlighted in red. The main corrections in the paper and the response to the reviewer’s comments point-by-point are as following:

**Reviewer #1:**

1. *For all the manuscript: a space between the last word of the sentence and the reference number must be respected.*

**Response:** Thank you for your professional suggestion and we have revised all the manuscript according to your suggestion.

2. *Methods section : Patients followed for motor disabilities such as cerebral palsy must be mentioned in the exclusion criteria section as they may be complicated by chest wall deformities.*

**Response:** Thanks for your helpful suggestions. We have incorporated your suggestions into the exclusion criteria section.

3. *Results section:
   -Line 179 : N% must be clarified and specified in the abbreviation section.
   -Line 181: sentence : and PCT (0.18 There……….. seems confused and incomplete.
   - Line 183: steroid application must be substituted by steroid injection or steroid treatment. 
   - Lines 191-192: "duration" barred (duration): must be corrected; the first "and" must be removed.*

**Response:** We sincerely apologize for our carelessness. We greatly appreciate your valuable comments and suggestions. We have now made the necessary corrections and have marked all the revised parts in red for your easy identification..

4. Discussion section:
   - *Line 214: BO must be written firstly in full before abbreviation and also incorporated in abbreviation list.*

**Response:**Thank you for bringing this important point to our attention. We have now made the necessary corrections in the manuscript.
 *- Line 245: the author report that longer fever durations lead to greater damage to the lung parenchyma; this idea must be more delopped with if possible more publications.*

**Response:**Thank you for your valuable suggestion. We have developed this section in the revised manuscript and highlighted in red.

*- Line 288 : "A study of 830 patients revealed that inflammatory indicators in288 children with NP in the acute phase were consistent with the level of pulmonary289 inflammation". This sentence or idea is unclear and inconsistent with the sentences before and after it.*

**Response:**Thank you very much for your valuable feedback. We feel sorry for the unclearness of the section and have now made the necessary corrections and have marked all the revised parts in red for your easy identification..
 *- Line 302: "changes in lung imaging….." : appear not clear, should be simplified.*

**Response:**Thank you very much for pointing out this issue. We have simplified and refined the relevant content .
 *- line 308 : the authors compare CRP levels with others studies (ref 10 and 18). This comparison must be made in two separate sentences.*

**Response:**Thank you for your valuable feedback. We truly appreciate your attention to the details of our manuscript. We have made the necessary revisions to ensure that the sentence more accessible and easier to understand.
 *- Line 327: "Children with NP have a long fever duration and significantly increased levels of inflammatory markers." This sentence needs to be put in a new paragraph for a good connection of ideas.*

**Response:**Thank you for your valuable feedback. We have carefully considered your suggestion and placed this sentence in a new paragraph.

**Reviewer #2:**

*At abstract section; rewrite conclusion section*

**Response:**Thank you for your constructive feedback.We have carefully reviewed and reworked the conclusion in the abstract to ensure that it accurately summarizes the key findings and implications of our study.
 *From line 98 to 102; write in separate section using Ethical consideration section at last of method part.*

**Response:**Thank you for your valuable suggestion. We have made the necessary adjustments based on your suggestion.
 *Write study area and study design*

**Response:**Thank you for your comment. We have rewrited the sections on the study area and study design as you suggested.

*Have you study sample or take all? If you take sample state?*

**Response:**We retrieved the data of all pediatric patients who were diagnosed with NP and had a comprehensive information between January 2018 and December 2019. Employing the random number table approach, 441 pediatric cases diagnosed with NNP during the same period were randomly recruited.
 *Why you take patients between the ages of 1 and 16 years and you take the season January 2018 to December 2019. Season has effect on pneumonia? So, you do not incorporate it, state it at limitation for further study.*

**Response:**Thank you for your comments. According to the research of our center (unpublished data), 2018 and 2019 were the epidemic years of MP infection in Tianjin, China. Season does have effect on pneumonia and winter (from November to January) is a season with a high incidence of pneumonia in Tianjin, China. Therefore, we selected the cases from January 2018 to December 2019, covering two calendar years. Based on the research conducted by our center (unpublished data), it was found that the positive rate of MP in children under 1 year old was only 3.42% and the clinical manifestations were not typical. Therefore, we only selected children with MPP aged 1 to 16 years and We state it at limitation part for further study.

Changes in the Detection Rate of MP among Children with CAPfrom January 2013 to December 2023

|  | 检测例数 | 阳性率 |
| --- | --- | --- |
| 2013年 | 1849 | 22.0119 |
| 2014年 | 916 | 16.7031 |
| 2015年 | 923 | 15.9263 |
| 2016年 | 5589 | 24.94 |
| 2017年 | 8121 | 25.1816 |
| 2018年 | 9742 | 25.4978 |
| 2019年 | 12445 | 27.8988 |
| 2020年 | 4523 | 10.0818 |
| 2021年 | 10178 | 9.18648 |
| 2022年 | 4894 | 11.8717 |
| 2023年 | 18941 | 22.4751 |

*State the limitation and strength of this study in separate section from discussion. State the strength of the study*

**Response:**Thank you for your valuable suggestion. We have made the necessary adjustments based on your suggestion.
 *How to select variables for multivariable logistic regression?*

**Response:**Thank you for your helpful comment. Variables significant (p<0.05) at univariate analysis were included in the multivariable model
 *In method part you have discuss about chi-square test, Mann-whitney U tests, fisher….. however, you did not do at result and why you use Mann‒Whitney U tests in this study?*

**Response:** Thank you for your valuable feedback. When the continuous variables do not conform to the normal distribution, we applied the Mann-Whitney U test for the examination. When the data were of the categorical (count) data type, the chi-square test was employed for statistical analysis.
 *From line 184 to 188, do you do Adjusted odds ratio or crude odds ratio, modify it.*

**Response:** Thank you for pointing out the issue and we sincerely appreciate your careful review. we would like to clarify that it is adjusted odds ratio
 *Write the conclusion again by including significant risk factors.*

**Response:**Thank you for your valuable suggestion. We have made the necessary adjustments based on your suggestion.

*What is the recommendation based on your study? Write it*

**Response:**Thank you for your helpful comments. In patients with RMPP, persistent high fever, along with marked elevations in WBC counts, IL- 6 levels, and D - dimer values may be the indicator of NP and pulmonary imaging changes should be closely monitored for the early detection of pulmonary necrosis

*Write the abbreviation in alphabet*
**Response:**Thank you for your valuable suggestion. We have made the necessary adjustments based on your suggestion.

***additional comments that were saved as attachments.***

*you have collect sex, age... retrospectively, however you have take sample, in addition, patients underwent diagnostic testing for complete blood count, .....*

**Response:**Thank you for your helpful comments. Those demographic information, including name, gender, age, as well as laboratory data and imaging characteristics, were all stored in the medical record system of our hospital upon the patients’ admission. When conducting this study, We retrieved and analyzed the data of pediatric patients who met the diagnostic criteria.

Additionly, the other issues within the attachments have also been revised in the article and the revised parts were all marked in red
